# Dr. John McBeth

**Published:** 1903-06

**Authors:** 


Dr. John McBeth, of Wales Center, N.Y., died at his home in that village, February 16, 1903, aged 80 years. He was born at Dalhousie, Can., September 25, 1822, of Scottish parentage.

He came to Wales Center in 1843, where he studied medicine with his brother, Dr. Gilbert McBeth. In 1846, the latter removed to Buffalo, leaving his practice in the hands of his brother John. Gilbert McBeth died on his way to California in 1849.

Dr. John McBeth, from the year 1846, until near the close of his life, continued to practise medicine at Wales Center, where he enjoyed a high reputation for professional skill and personal integrity. He was elected to the board of supervisors in 1860, and served as a member for two terms. He became assistant surgeon of the 67th regiment, N.Y.N.G., and served with his command in the Gettysburg campaign of 1863. Dr. McBeth married Clara P. Tabor, August 11, 1871, and she died February 23, 1876. He did not marry again and is survived by an only child, Mrs. William Foss, of Wales Center, and a brother, James McBeth, of Illinois. A sister, Mrs. Helen Tobias, of Buffalo, has died since the decease of Dr. McBeth.

